# Endoscopy and noninvasive tests in pediatric disorders of gut–brain interaction: A multicenter retrospective study of the Italian Society of Pediatric Gastroenterology, Hepatology, and Nutrition

**DOI:** 10.1002/jpn3.70167

**Published:** 2025-07-21

**Authors:** Mattia Spatuzzo, Chiara Imondi, Paola De Angelis, Fortunata Civitelli, Valentina Giorgio, Caterina Strisciuglio, Renato Tambucci, Federica Ferrari, Cosimo Ruggiero, Giuseppina Russo, Danilo A. Fegatelli, Salvatore Oliva

**Affiliations:** ^1^ Women's and Children's Health Department, Pediatric Gastroenterology and Hepatology Unit Sapienza University of Rome Rome Italy; ^2^ Gastroenterology and Nutrition Unit, Bambino Gesù Children's Hospital Rome Italy; ^3^ Department of Gender Diseases Child and Adolescent Health, Pediatric Unit, Sant'Eugenio Hospital Rome Italy; ^4^ Department of Translational Medicine Gastroenterology and Hepatology Unit Sapienza University of Rome Rome Italy; ^5^ Department of Woman and Child Health and Public Health, Fondazione Policlinico Universitario “A. Gemelli” IRCCS Università Cattolica del Sacro Cuore Rome Italy; ^6^ Department of Woman, Child and General and Specialistic Surgery University of Campania “Luigi Vanvitelli” Naples Italy; ^7^ Department of Life Sciences, Health and Health Professions Link Campus University Rome Italy

**Keywords:** pediatric DGBIs, red flags, Rome IV criteria

## Abstract

**Objectives:**

Disorders of gut–brain interactions (DGBIs) are highly prevalent in pediatric gastroenterology and often lead to the use of invasive and noninvasive diagnostic tests, despite the guidance provided by the Rome IV criteria. Rome IV promotes a positive diagnostic approach based on the identification of specific symptoms occurring at defined frequencies, unexplained after thorough medical evaluation. This study aimed to evaluate the frequency and diagnostic accuracy of these tests in pediatric DGBIs.

**Methods:**

We conducted a retrospective analysis of pediatric patients (aged 2–16 years) evaluated for suspected DGBIs, as defined by the Rome IV criteria, across five national pediatric gastroenterology centers. Patients with known underlying organic gastrointestinal diseases were excluded. Patients were grouped based on the presence or absence of “red flags” for organic pathology, including a positive family history for gastrointestinal diseases, persistent pain outside the periumbilical area, nocturnal symptoms, persistent, bilious, or bloody vomiting; presence of blood in stools or anemia; unexplained fever; altered growth parameters, delayed or abnormal puberty; associated gastrointestinal or extraintestinal symptoms; and abnormal physical findings. Clinical data, final diagnoses, and all performed tests were recorded. Statistical analysis assessed the frequency and diagnostic accuracy of each test, and a multivariate model developed to improve diagnostic accuracy.

**Results:**

We included 500 patients with suspected DGBIs from five centers: 52.5% were female, and the median age was 9.1 ± 4.5 years. Red flags were present in 45% of patients, showing a higher frequency of positive family history, elevated inflammatory serological markers, and fecal calprotectin (FC) levels. Patients with red flag underwent more endoscopies; however, no significant increase in the detection of organic disease was observed. No single test alone demonstrated sufficient accuracy in predicting organic pathology. A multivariate model combining the presence of red flags, positive family history, elevated serum platelet count, and increased FC achieved the highest accuracy (area under the curve: 0.711, 95% confidence interval: 0.63–0.79).

**Conclusions:**

A model combining red flags for organic disease, positive family history, elevated serum platelet count, and increased FC may aid in the identification of organic diseases in children with suspected DGBIs. Prospective studies are needed to validate this model and to support the diagnostic process when Rome IV criteria alone do not distinguish between organic and functional disorders.

## Abstract (highlights)

**What is Known:**

- The Rome IV criteria recommend a positive diagnostic approach for disorders of gut–brain interactions (DGBIs); however, clinical practice frequently involves laboratory and instrumental testing of uncertain utility.
- No standardized diagnostic work‐up currently exists for DGBIs.
- Patients with DGBIs often undergo unnecessary invasive endoscopic procedures, increasing the diagnostic burden on patient and their families. This practice may also detract from a necessary psychosomatic approach to care.

**What is New:**

- We evaluated the real‐world application of the Rome IV criteria in Italy, assessing the utility of laboratory and instrumental tests in pediatric DGBIs.
- In our large cohort of patients with suspected DGBIs, over 80% maintained a functional diagnosis based solely on clinical history, even after diagnostic testing, supporting the Rome IV approach centered on clinical evaluation. Fewer than 20% received a final diagnosis of organic disease; among these, only two‐thirds exhibited red flags at onset, suggesting that red flags alone have limited diagnostic accuracy and require integration with additional clinical and laboratory parameters.
- Despite undergoing a higher number of invasive tests, patients with red flags did not show a statistically significant increase in endoscopically confirmed organic pathology.

## Graphical abstract

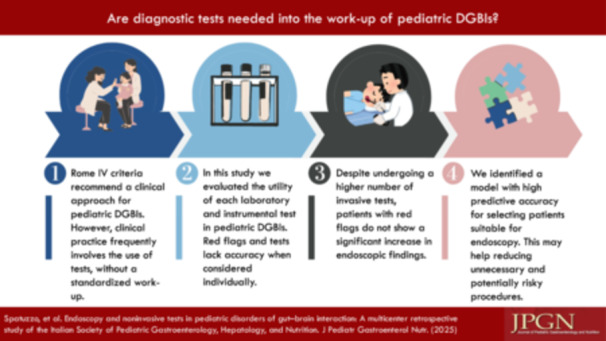

## 1 INTRODUCTION

Disorders of gut–brain interactions (DGBIs) are common in the pediatric population. They refer to a group of conditions with a wide spectrum of manifestations, characterized by gastrointestinal dysmotility, visceral hypersensitivity, microbiome alterations, mast cell involvement, altered central nervous system processing, and local mucosal changes.^1^ However, their exact pathophysiology remains incompletely understood.^2^

**Figure 1:**
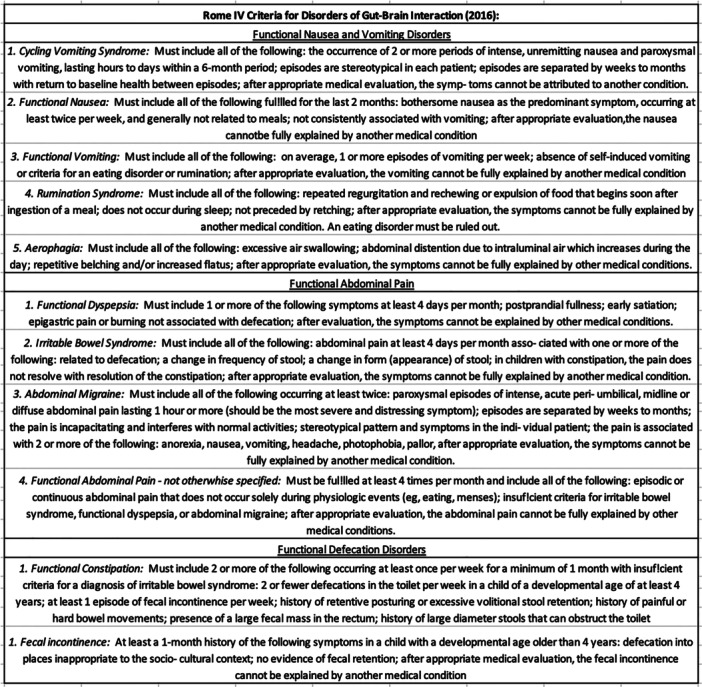
Rome IV criteria for the clinical diagnosis of DGBIs (2016). DGBIs, disorders of gut–brain interactions.

The conceptual framework for DGBIs has evolved from a psychosomatic to a biopsychosocial model, acknowledging psychological and social influences.^3^, ^4^ To support clinical diagnosis, the Rome Committee developed criteria specifying necessary symptoms and recurrence intervals for each disorder (Figure 1).^5^ According to Rome IV criteria, DGBIs are grouped into three main categories: functional nausea and vomiting disorders, functional abdominal pain disorders (e.g., irritable bowel syndrome [IBS], functional dyspepsia), and functional defecation disorders (e.g., constipation, fecal incontinence).

Although Rome IV promotes a symptom‐based diagnosis¹, clinical practice often focuses on excluding alarm signs of organic disease. The presence of red flags precludes a purely functional diagnosis and requires further work‐up.

Several challenges underscore the need for focused research in pediatric DGBIs. First, DGBIs represent one of the most common reasons for pediatric gastroenterology consultations, with global estimates suggesting that more than 25% of children fulfill diagnostic criteria.^6^, ^7^ Second, despite recommendations supporting a symptom‐based approach, clinical practice often includes extensive diagnostic testing, which can cause unnecessary discomfort and increased costs.^8^ Moreover, there is no standardized diagnostic pathway, leading to inconsistent management and potential overuse of invasive procedures. Third, the economic burden is significant: UK data estimate annual management costs for pediatric DGBIs at £73 million,^9^ while US estimates suggest over $11,787 per adolescent annually, excluding indirect costs such as missed school or parental work absenteeism.^10^

In this multicenter retrospective study, promoted by the Italian Society of Pediatric Gastroenterology, Hepatology, and Nutrition (SIGENP), we aimed to evaluate the diagnostic utility of key clinical, laboratory, and imaging tests in pediatric DGBIs. Primary goals were to assess the frequency of organic versus functional diagnoses and determine the diagnostic accuracy of clinical history, labs, and imaging. Finally, we aimed to build a multivariate logistic model combining clinical and laboratory variables to guide appropriate use of invasive investigations.

## 2 METHODS

### 2.1 Ethics statement

The study was approved by the Ethics Committee of Policlinico Umberto I, Rome, which served as the coordinating center (Protocol No. 0045/2025).

### 2.2 Study design and population

This retrospective, multicenter observational study included pediatric patients aged 2–16 years evaluated for suspected DGBIs, according to Rome IV criteria, between January 2020 and May 2022. Patients younger than 2 years or older than 16 years, those with known organic disease, or presenting for unrelated conditions were excluded (Figure S1).

### 2.3 Participating centers

Five Italian pediatric referral centers participated: Policlinico Umberto I in Rome; Bambino Gesù Children's Hospital in Rome; Gemelli University Hospital Foundation in Rome; Sant' Eugenio Hospital in Rome; and the University of Campania “Luigi Vanvitelli” in Naples. Inclusion criteria were age eligibility and clinical suspicion of DGBIs according to Rome IV criteria (Figure 1). Patients with known gastrointestinal diseases (e.g., food allergies) were excluded (Figure S1).

### 2.4 Red flags

Patients were stratified based on the presence of red flags for organic pathology, defined according to Rome IV: family history of gastrointestinal (GI) disease, persistent pain localized outside the periumbilical region, particularly in the right quadrants, nocturnal symptoms, persistent or bilious vomiting, hematemesis or blood in stools, anemia, unexplained fever, growth failure, delayed puberty, dysphagia, perianal disease, arthritis, or palpable masses.

### 2.5 Data collection

In both groups, collected data included demographics, clinical features, anthropometric measurements, suspected diagnoses, diagnostic tests, comorbidities, therapies, and final diagnoses.

### 2.6 Serum laboratory parameters

Laboratory values were evaluated using standard reference thresholds: hemoglobin (Hb) 12.1 g/dL and platelet count (PLT) 430.000/L. Erythrocyte sedimentation rate (ERS) and C‐reactive protein (CRP) were considered pathological if >25 mm/h and 0.6 mg/dL, respectively. Fecal calprotectin (FC) values were expressed in μg/g, with a positive test defined as >150 μg/g. Celiac screening was performed using tissue‐transglutaminase‐immunoglobulin A (IgA), with <20 AU/mL considered negative.

### 2.7 Instrumental tests

Abdominal ultrasound and digestive endoscopy were performed across centers. Biopsies were processed per national guidelines by experienced pathologists.

### 2.8 Primary and secondary outcomes

The primary objective was to evaluate the accuracy of each test in predicting organic pathology, assessed by receiver operating characteristic (ROC) curves for demographic variables (age, gender, family history of GI disease, and presence of red flags), laboratory indices (white blood cells [WBCs], PLT, ERS, CRP, and FC), and diagnostic tests (abdominal ultrasound, esophagogastroduodenoscopy [EGD], ileocolonoscopy). Additionally, we sought to identify the most effective combination of diagnostic and clinical variables to develop a multivariate logistic regression model aimed at improving the selection of patients for invasive procedures.

### 2.9 Statistical analysis

Statistical analysis was performed using R software (v 3.5.1). chi‐square tests were used for categorical variables and the Mann–Whitney *U* test (Wilcoxon rank‐sum test) for continuous variables. In Table 2, results are presented as median (interquartile range) or mean (standard deviation). Area under the curve (AUC) values for ROC analysis were interpreted as follows: AUC = 0.5 not informative, 0.5 < AUC < 0.7 low accurate, 0.7 < AUC < 0.9 moderate accuracy, 0.9 < AUC < 1.0 high accuracy, AUC = 1 perfect test.

## 3 RESULTS

### 3.1 Demographic data

Our study included 500 patients with suspected DGBIs (Figure S1): 52.5% were female and of school age, with a median age of 9.1 ± 4.5 years. Patients were divided into two groups based on the presence or absence of one or more red flags for organic pathology at initial assessment: 45% of the cohort presented at least one red flag. The frequency of red flags is detailed in Table 1.

**Table 1:** Demographic information, presence of red flags, family history, comorbidities, diagnostic suspicion at the onset, diagnosis at discharge, and therapy.

	Overall	No red flags	Red flags	*p*
Demographic characteristics				
Number of patients	500	272	228	
Gender (Male)	237	125	112	0.563
Age at initial evaluation	9.1	8.4	10	<0.001
Diagnostic suspicion (according to Rome IV)				
Recurrent abdominal pain	43.9% (219)	35.8% (97)	53.5 (122)	<0.001
Cyclic vomiting syndrome	1.2% (6)	0.7% (2)	1.8% (4)	0.532
Functional diarrhea	12% (60)	9.2% (25)	15.4% (35)	0.05
Functional constipation	40.7 (203)	46.5% (126)	33.8% (77)	0.005
Functional nausea and vomiting	21.8% (109)	21.8% (59)	21.9% (50)	1.000
IBS	4.4% (22)	5.5% (15)	3.1% (7)	0.264
Abdominal migraine	0.6% (3)	1.1% (3)	0 (0)	0.311
Red flags (according to Rome IV)				
Localized pain	9.2% (46)	0	20.2% (46)	N.A.
Nocturnal pain	8.4% (42)	0	18.4% (42)	N.A.
Weight loss	5.2% (26)	0	11.4% (26)	N.A.
Perianal disease	1.4% (7)	0	3.1% (7)	N.A.
Dysphagia	4% (20)	0	8.8% (20)	N.A.
Persistent vomiting	1.4% (7)	0	3.1% (7)	N.A.
Growth deceleration	2.8% (14)	0	6.1% (14)	N.A.
Delayed puberty	0	0	0	N.A.
Rectal bleeding	12% (60)	0.4% (1)	25.9% (59)	N.A.
Nocturnal diarrhea	0.8% (4)	0	3.5% (8)	N.A.
Fever	1.6% (8)	0	1.8% (4)	N.A.
Family history of GI disorders	11.6% (58)	0.4% (1)	25% (57)	N.A.
Comorbidities				
Personal comorbidities (all)	42.9% (214)	41.7% (113)	44.3% (101)	0.621
Peptic ulcer disease	2% (10)	2.2% (6)	1.8% (4)	0.965
Cardiovascular diseases	2.6% (13)	3.3% (9)	1.8% (4)	0.417
Epilepsy/neurological disorders	5.8% (29)	6.3% (17)	5.3% (12)	0.773
Migraine	4.8% (24)	4.4% (12)	5.3% (12)	0.823
Allergic conditions	17.4% (87)	16.2% (44)	18.9% (43)	0.515
Rheumatological diseases	2.4% (12)	1.1% (3)	3.9% (9)	0.077
Psychiatric disorders	3.2% (16)	3.7% (10)	2.6% (6)	0.679
Obesity	1.8% (9)	1.8% (5)	1.8% (4)	1.000
Endocrine disorders	3.6% (18)	2.2% (6)	5.3% (12)	0.114
Hepato‐pancreatic diseases	2.2% (11)	1.8% (5)	2.6% (6)	0.772
Renal diseases	1% (5)	0.7% (2)	1.3% (3)	0.846
Family history				
Family history of GI disorders (all)	29.5% (147)	20.3% (55)	40.4% (92)	<0.001
IBD	4.6% (23)	1.5% (4)	8.3% (19)	0.001
Celiac disease	5% (25)	1.1% (3)	9.6% (22)	<0.001
EOE	0.4% (2)	0 (0)	0.9% (2)	0.404
Peptic ulcer disease	10.4% (52)	8.5% (23)	12.7% (29)	0.163
Migraine	1.2% (6)	0.4% (1)	1.8% (4)	0.199
Hepato‐pancreatic disorders	1% (5)	0.4% (1)	1.8% (4)	0.199
Allergic conditions	6% (30)	3.3% (9)	9.2% (21)	0.01
Diagnosis at discharge				
Functional abdominal pain	21.7% (108)	19.3% (52)	24.6% (56)	0.186
Cyclic vomiting syndrome	1.8% (9)	1.8% (5)	1.8% (4)	1.000
Functional constipation	42.8% (213)	47.4% (128)	37.3% (85)	0.029
Functional diarrhea	3.2% (16)	3.3% (9)	3.1% (7)	1.000
Functional nausea and vomiting	8% (40)	8.9% (24)	7% (16)	0.557
IBS	9.4% (47)	10% (27)	8.8% (20)	0.764
IBD	2.2% (11)	1.1% (3)	3.5% (8)	0.13
Celiac disease	1.8% (9)	1.1% (3)	2.6% (6)	0.349
GERD	10.8% (54)	8.1% (22)	14% (32)	0.048
*Helicobacter pylori* infection	1.2% (6)	1.1% (3)	1.3% (3)	1.000
Hepato‐pancreatic disease	0.6% (3)	0 (0)	1.3% (3)	0.189
Abdominal migraine	0.8% (4)	0.7% (2)	0.9% (2)	0.838
Therapies				
Probiotics	32.1% (160)	34.7% (94)	28.9% (66)	0.203
Antispasmodics	14.6% (73)	11.1% (30)	18.9% (43)	0.02
Antidiarrheals	0.4% (2)	0.4% (1)	0.4% (1)	1.000
Tricyclic antidepressant	1.2% (6)	1.1% (3)	1.3% (3)	1.000
Prokinetics	2.8% (14)	2.2% (6)	3.5% (8)	1
Laxatives and stool softeners	47.3% (236)	49.8% (135)	44.3% (101)	0.254
PPIs	20.4% (102)	13.7% (37)	28.5% (65)	<0.001
Alginates	21.8% (109)	18.1% (49)	26.3% (60)	0.035
Cyproheptadine	1.2% (6)	1.1% (3)	1.3% (3)	1
Mesalazine	2.2% (11)	1.1% (3)	3.5% (8)	0.13
Antibiotics	6.8% (34)	5.9% (16)	7.9% (18)	0.483

Abbreviations: EOE, eosinophilic esophagitis; GERD, gastroesophageal reflux disease; GI, gastrointestinal; IBD, inflammatory bowel disease; IBS, irritable bowel syndrome; N.A., not applicable; PPI, proton pump inhibitors.

### 3.2 Initial suspicion and final diagnosis

At presentation, the most frequent clinical suspicion was functional abdominal pain not otherwise specified (FAP‐NOS), especially among those with red flags (53.5%, *p* < 0.001), while functional constipation was more common in patients without red flags (46.5%, *p* = 0.005). At discharge, 81% received a confirmed DGBI diagnosis—most commonly functional constipation (42.8%) and FAP‐NOS (21.7%). Only 19% were diagnosed with organic pathology, mainly peptic ulcer disease and GERD (10.8%), and just 62% of these had red flags initially (*p* = 0.001). Further details on final diagnoses are provided in Table 1.

### 3.3 Comorbidities

Overall, 43% of the cohort had at least one comorbidity, most commonly allergic conditions (17.4%), neurological disorders (5.8%; *n* = 29), and headaches (4.8%; *n* = 12) (Table 1). There was no significant difference in the prevalence of comorbidities between the groups with and without red flags.

### 3.4 Laboratory tests

All patients underwent laboratory investigations, including complete blood count and inflammatory markers. Celiac serology was conducted in 59% of the cohort. No significant differences emerged in CRP, Hb, or tissue‐transglutaminase‐IgA levels (Table 2). However, FC levels were higher in the red flag group (median 35 μg/g vs. 15.6 μg/g; *p* < 0.001). Similar findings were observed for ERS and WBC count.

**Table 2:** Frequency of the use of each test in the entire cohort and between the groups with and without red flags.

	Overall	No red flags	Red flags	*p*
Laboratory investigations				
Total Hb (g/dL)	13.0 (12.0; 13.8)	13.0 (12.0;13.8)	12.9 (12.2; 13.7)	0.461
ERS (mm/h)	2 (0;7)	2 (0;5)	1 (0;5)	0.4
CRP (mg/dL)	0.06 (0.02; 0.2)	0.06 (0.02; 0.2)	0.07 (0.03; 0.32)	0.949
WBCs (x10^3^)	6900 (5400; 8180)	6705 (5400; 8102)	7100 (5750; 8300)	0.383
PLTs (x10^3^)	282,000 (235,000; 316,000)	280,000 (241,000; 318,000)	285,000 (228,750; 311,500)	0.065
Fecal calprotectin (μg/g)	23.9 (1; 38.3)	15.6 (1; 36.2)	35.0 (24.0; 47.2)	<0.001
Celiac screening conducted	59.3% (296)	57.6% (156)	61.4% (140)	0.436
Positive celiac screening	1.8% (9)	1.1% (3)	2.6% (6)	0.349
Instrumental investigations				
Ultrasound performed	19% (95)	22% (60)	15.4% (35)	0.07
Abnormal ultrasound findings	4.4% (22)	2.2% (6)	7% (16)	0.017
pH metry/impedance testing conducted	3% (15)	2.6% (7)	3.5% (8)	0.734
Abnormal pH metry/impedance testing	0.4% (2)	0.7% (2)	0 (0)	0.556
Performed EGD	20% (100)	12.5% (34)	28.9% (66)	<0.001
Abnormal EGD	5.6% (28)	4.1% (11)	7.5% (17)	0.148
Abnormal histology in EGD	4.6% (23)	2.6% (7)	7% (16)	0.032
Colonoscopy performed	10% (50)	4.1% (11)	17.1% (39)	<0.001
Abnormal colonoscopy findings	0.8% (4)	1.1% (3)	0.4% (1)	0.741
Abnormal histology in colonoscopy	1.6% (8)	1.5% (4)	7% (16)	1.000
Other diagnostic tests performed				
Total	50.3% (251)	46.1% (125)	55.3% (126)	0.052
Entero‐MRI	2.2% (11)	0.4% (1)	4.4% (10)	0.006
Brain‐MRI	1.4% (7)	1.1% (3)	1.8% (4)	0.818
Anorectal manometry	2.2% (11)	3% (8)	1.3% (3)	0.35
Esophageal manometry	0 (0)	0 (0)	0 (0)	0.054
Upper GI series (barium swallow)	2.8% (14)	2.2% (6)	3.5% (8)	0.548
Sweat chloride testing	1.2% (6)	1.5% (4)	0.9% (2)	0.842
Allergy testing	13.6% (68)	12.9% (35)	14.5% (33)	0.708
Stool culture and parasitological tests	11.8% (59)	8.9% (24)	15.4% (35)	0.036
*Helicobacter pylori* detection	11.2% (56)	10.3% (28)	12.3% (28)	0.586
Abnormal abdominal X‐ray	4.6% (23)	5.2% (14)	3.9% (9)	0.665
Thyroid screening conducted	23.6% (118)	17.7% (48)	30.7% (70)	0.001
Lactose breath test	3.4% (17)	2.2% (6)	4.8% (11)	0.178

*Note*: Quantitative variables are presented as median (IQR), qualitative variables as mean (SD).

Abbreviations: CRP, C‐reactive protein; EGD, esophagogastroduodenoscopy; ERS, erythrocyte sedimentation rate; GI, gastrointestinal; Hb, haemoglobin; IQR, interquartile range; MRI, magnetic resonance imaging; PLTs, platelets; SD, standard deviation; WBCs, white blood cells.

### 3.5 Instrumental tests

Endoscopy was performed in 150 patients (100 EGD, 50 ileocolonoscopy), more frequently in those with red flags, though this did not correlate with more organic diagnoses (*p* = 0.148). Additional diagnostic tests were performed in 50.3% of patients, with MR‐enterography, stool microbiology, and thyroid function tests showing differences between groups. Table 1 summarizes the frequency and diagnostic impact of the tests performed.

### 3.6 Medical therapies

The most prescribed treatments were laxatives (47%), probiotics (32.1%), and proton pump inhibitors (PPIs) or alginates (20%). Patients with red flags received more therapies, particularly PPIs (*p* < 0.01), antispasmodics (*p* = 0.02), and alginates (*p* = 0.035).

### 3.7 Diagnostic tests accuracy

**Figure 2:**
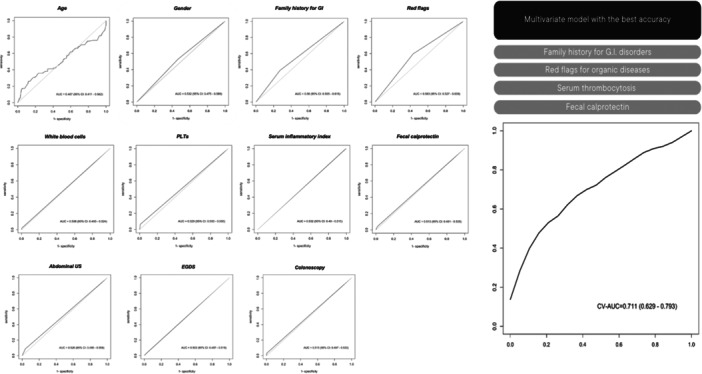
Accuracy of each diagnostic test and of the best multiple logistic combination to predict a final diagnosis for organic disease in our work. Egd, Esophagogastroduodenoscopy; GI, Gastro‐intestinal; Plts, Platelets; Us, Ultrasound.

ROC analysis of individual variables did not show sufficient accuracy for predicting organic disease (Figure 2). Therefore, a multiple logistic regression model combining clinical and laboratory variables was developed (Figure S2). The most accurate combination included red flags, FC, thrombocytosis, and family history of GI disease showed the best performance (AUC: 0.711; 95% confidence interval [CI]: 0.63–0.79). (odds ratio [OR] red flags 1.289, 95% CI: 0.749–2.170, OR PLTs 2.570, 95% CI: 1.475–4.636; OR FC 1.027, 95% CI: 1.004–1.057; OR family history of GI disease GI 2.020, 95% CI: 0.895–4.548) (Figure 2).

## 4 DISCUSSION

In this multicenter retrospective study, we aimed to evaluate the utility of diagnostic tests in the work‐up of pediatric DGBIs by analyzing their frequency and diagnostic accuracy. Most patients ultimately received a functional diagnosis, with diagnostic tests often confirming the initial clinical suspicion, suggesting that many investigations may be unnecessary in the absence of alarm features. Conversely, only 19% had organic pathology, and just 62% of these showed red flags at presentation—indicating that red flags alone are not sufficiently accurate for predicting organic disease. To improve diagnostic accuracy, we explored combinations of clinical features and tests. While patients with red flags more often had abnormal findings (Figure S3), no single marker reliably distinguished organic from functional disorders.

FC, a noninvasive inflammation marker, has gained attention. Though limited by handling and reference values, both ESPGHAN^11^ and the Rome Foundation^12^ recommend FC to help distinguish between functional gastrointestinal disorders and inflammatory bowel diseases (IBD). Despite the higher diagnostic performance in children compared to adults, there is no universally accepted cut‐off,^13^ we used 150 μg/g, a value supported by literature as balancing sensitivity and specificity. In our cohort, FC levels alone did not differ significantly between patients with and without red flags. However, its diagnostic utility improved when combined with other clinical variables.^14^

Regarding *celiac screening,* previous studies show higher prevalence in children with IBS‐like symptoms.^15^, ^16^ Based on this, Rome IV recommends screening in all children with suspected IBS.^17^ In our cohort, testing was frequent, particularly in suspected functional abdominal pain (73%), functional diarrhea (90%), and functional constipation (46%). However, diagnostic yield remained low, and no clinical predictors for screening emerged.

Despite literature supporting selected use^18^, ^19^ our study found no significant differences in diagnostic yield from other tests—including abdominal ultrasound, pH‐impedance, abdominal or digestive tract X‐rays, manometry, breath tests, or MRI. Despite their common use, the Rome criteria do not recommend routine *abdominal ultrasound* due to its low specificity. A 2019 review highlighted its limited diagnostic value, noting it primarily serves to reassure families and clinicians.^12^

Endoscopy presents a particular challenge. ESPGHAN recommends it only in the presence of red flags, though the evidence is limited.^17^ One US study found that among 290 children undergoing EGD for suspected functional symptoms, only 38% had a conclusive organic diagnosis. An Australian study similarly reported that only 10% of colonoscopies revealed organic pathology, usually in patients with prior abnormal inflammatory markers.^20^ These findings suggest that history and noninvasive biomarkers may better identify candidates for invasive procedures. Still, endoscopy is crucial for avoiding delayed diagnoses of serious conditions such as IBD and celiac disease, which can present with functional‐like symptoms. About 30%–40% of children with IBD or celiac disease initially present with functional complaints.^21^, ^22^ Early endoscopy can expedite diagnosis and improve outcomes, particularly in children whose growth is affected. Economically, while avoiding endoscopy may seem cost‐effective, repeated noninvasive testing may ultimately raise healthcare costs. Yet, as seen in previous studies, endoscopy often fails to significantly impact management in suspected DGBIs unless guided by risk stratification.^23^, ^24^, ^25^ One proposed strategy involves combining clinical and laboratory markers. For example, a risk‐stratified model by Mark et al.^26^ led to a much lower rate of organic diagnoses in low‐risk patients (6%) compared to high‐risk ones (34%).

Inspired by this, we aimed to identify the most effective individual test or *combination of tests* for selecting patients who might benefit from endoscopy. ROC analysis showed that no single variable had sufficient predictive value. However, multivariate analysis revealed that a combination of red flags, FC, thrombocytosis, and a positive family history of gastrointestinal disease provided the best diagnostic accuracy (AUC: 0.711; 95% CI: 0.63–0.79).

*Therapeutic management* showed no major differences between groups. Laxatives, PPIs, antispasmodics, alginates, and probiotics were commonly prescribed across both functional and organic diagnoses, indicating overlapping treatment strategies in clinical practice.

*This study has several* limitations. Its retrospective design may introduce bias, however, the large sample and inclusion of five centers reflect real‐world variability. Second, a standardized diagnostic protocol was not applied across centers, however, given the lack of standardized protocols for DGBIs, our findings reflect real‐world practice. Third, we did not differentiate between DGBI subtypes, although functional gastrointestinal disorders frequently overlap in pediatric patients, making strict subclassification challenging. Finally, our multivariate model has not yet been prospectively validated. To address this, we have initiated a prospective study enrolling a larger cohort of patients with suspected DGBIs.

## 5 CONCLUSION

In our cohort, red flags alone were insufficient to reliably identify candidates for invasive procedures. No single first‐line test predicted organic disease with high accuracy. However, a combination of red flags, family history, thrombocytosis, and elevated FC levels was associated with higher odds of organic pathology, supporting more extensive investigations in selected patients. Further prospective studies are needed to validate this approach, particularly when Rome IV criteria alone are inadequate. If confirmed, this model may help reduce unnecessary procedures while improving diagnostic efficiency in children with suspected DGBIs.

## CONFLICT OF INTEREST STATEMENT

The authors declare no conflict of interest.

## Supporting information


**Supplementary fig. 1** Study design and population.

**Supplementary fig. 2** Univariate and multivariate analysis of the examined variables for predicting the presence of an organic disorder.

**Supplementary fig. 3** Comparison between groups with and without red flags for each laboratory test.

## References

[1] Drossman DA , Hasler WL . Rome IV‐functional GI disorders: disorders of gut‐brain interaction. Gastroenterology. 2016;150(6):1257‐1261. 27147121 10.1053/j.gastro.2016.03.035

[2] Zia JK , Lenhart A , Yang PL , et al. Risk factors for abdominal pain‐related disorders of gut‐brain interaction in adults and children: a systematic review. Gastroenterology. 2022;163(4):995‐1023.e3. 35716771 10.1053/j.gastro.2022.06.028 PMC9509486

[3] Van Oudenhove L , Levy RL , Crowell MD , et al. Biopsychosocial aspects of functional gastrointestinal disorders: how central and environmental processes contribute to the development and expression of functional gastrointestinal disorders. Gastroenterology. 2016;150:1355‐1367.e2. 10.1053/j.gastro.2016.02.027 PMC8809487 27144624

[4] Spatuzzo M , Chiaretti A , Capossela L , Covino M , Gatto A , Ferrara P . Abdominal pain in children: the role of possible psychosocial disorders. Eur Rev Med Pharmacol Sci. 2021;25(4):1967‐1973. 33660807 10.26355/eurrev_202102_25097

[5] Rasquin A , Di Lorenzo C , Forbes D , et al. Childhood functional gastrointestinal disorders: child/adolescent. Gastroenterology. 2006;130(5):1527‐1537. 16678566 10.1053/j.gastro.2005.08.063 PMC7104693

[6] Saps M , Velasco‐Benitez CA , Langshaw AH , Ramírez‐Hernández CR . Prevalence of functional gastrointestinal disorders in children and adolescents: comparison between Rome III and Rome IV criteria. J Pediatr. 2018;199:212‐216. 29747935 10.1016/j.jpeds.2018.03.037

[7] Robin SG , Keller C , Zwiener R , et al. Prevalence of pediatric functional gastrointestinal disorders utilizing the Rome IV criteria. J Pediatr. 2018;195:134‐139. 29398057 10.1016/j.jpeds.2017.12.012

[8] Drossman DA . Functional gastrointestinal disorders: history, pathophysiology, clinical features and Rome IV. Gastroenterology. 2016;150:1262‐1279.e2. 10.1053/j.gastro.2016.02.032 27144617

[9] Mahon J , Lifschitz C , Ludwig T , et al. The costs of functional gastrointestinal disorders and related signs and symptoms in infants: a systematic literature review and cost calculation for England. BMJ Open. 2017;7(11):e015594. 10.1136/bmjopen-2016-015594 PMC5695302 29138194

[10] Groenewald CB , Essner BS , Wright D , Fesinmeyer MD , Palermo TM . The economic costs of chronic pain among a cohort of treatment‐seeking adolescents in the United States. J Pain. 2014;15(9):925‐933. 24953887 10.1016/j.jpain.2014.06.002 PMC4150826

[11] Konincks CR , Donat E , Benninga MA , et al. The use of fecal calprotectin testing in paediatric disorders: a position paper of the European Society of Paediatric Gastroenterology and Nutrition, Gastroenterology Committee. J Pediatr Gastroenterol Nutr. 2021;72(4):617‐640. 10.1097/MPG.0000000000003046. 33716293

[12] Llanos‐Chea A , Saps M . Utility of diagnostic tests in children with functional abdominal pain disorders. Gastroenterol Hepatol. 2019;15(8):414‐422. PMC6771033 31592242

[13] Carroccio A , Iacono G , Cottone M , et al. Diagnostic accuracy of fecal calprotectin assay in distinguishing organic causes of chronic diarrhea from irritable bowel syndrome: a prospective study in adults and children. Clin Chem. 2003;49(6 Pt 1):861‐867. 12765980 10.1373/49.6.861

[14] Licata A , Randazzo C , Cappello M , et al. Fecal calprotectin in clinical practice: a noninvasive screening tool for patients with chronic diarrhea. J Clin Gastroenterol. 2012;46(6):504‐508. 22565607 10.1097/MCG.0b013e318248f289

[15] Cristofori F , Fontana C , Magistà A , et al. Increased prevalence of celiac disease among pediatric patients with irritable bowel syndrome a 6‐year prospective cohort study. JAMA Pediatr. 2014;168:555‐560. 10.1001/jamapediatrics.2013.4984 24756157

[16] Irvine AJ , Chey WD , Ford AC . Screening for celiac disease in irritable bowel syndrome: an updated systematic review and meta‐analysis. Am J Gastroenterol. 2017;112:65‐76. 10.1038/ajg.2016.466 27753436

[17] Thomson M , Tringali A , Dumonceau JM , et al. Paediatric gastrointestinal endoscopy: European Society for Paediatric Gastroenterology Hepatology and Nutrition and European Society of Gastrointestinal Endoscopy Guidelines. J Pediatr Gastroenterol Nutr. 2017;64:133‐153. 27622898 10.1097/MPG.0000000000001408

[18] Baumann‐Durchschein F , Fürst S , Hammer HF . Practical application of breath tests in disorders of gut‐brain interaction. Curr Opin Pharmacol. 2022;65:102244. 10.1016/j.coph.2022.102244 35636383

[19] Soliman H , Wuestenberghs F , Desprez C , Leroi AM , Melchior C , Gourcerol G . Alterations in gastrointestinal motility assessed by high‐resolution antroduodenal manometry in patients with severe disorders of gut‐brain interaction. Am J Physiol Gastrointest Liver Physiol. 2024;327(2):G306‐G315. 38860287 10.1152/ajpgi.00039.2024

[20] Kawada PS , O'Loughlin EV , Stormon MO , Dutt S , Lee CH , Gaskin KJ . Are we overdoing pediatric lower gastrointestinal endoscopy? J Pediatr Gastroenterol Nutr. 2017;64:898‐902. 26960173 10.1097/MPG.0000000000001192

[21] Colombel JF , Shin A , Gibson PR . AGA clinical practice update on functional gastrointestinal symptoms in patients with inflammatory bowel disease: expert review. Clin Gastroenterol Hepatol. 2019;17:380‐390.e1. 30099108 10.1016/j.cgh.2018.08.001 PMC6581193

[22] Silvester JA , Graff LA , Rigaux L , et al. Symptoms of functional intestinal disorders are common in patients with celiac disease following transition to a gluten‐free diet. Dig Dis Sci. 2017;62:2449‐2454. 28687943 10.1007/s10620-017-4666-z PMC5738027

[23] Oliva S , Pennazio M , Cohen SA . Capsule endoscopy followed by single balloon enteroscopy in children with obscure gastrointestinal bleeding: a combined approach. Dig Liver Dis. 2015;47(2):125‐130. 10.1016/j.dld.2014.09.001. 25266487

[24] Thakkar K , Chen L , Tatevian N , et al. Diagnostic yield of oesophagogastroduodenoscopy in children with abdominal pain. Aliment Pharmacol Ther. 2009;30:662‐669. 19573168 10.1111/j.1365-2036.2009.04084.x PMC3018747

[25] Bonilla S , Deli Wang U , Saps M . The prognostic value of obtaining a negative endoscopy in children with functional gastrointestinal disorders. Clin Pediatr. 2011;50:396‐401. 10.1177/0009922810392773 21242200

[26] Mark JA , Campbell K , Gao D , Kramer RE . Algorithm to predict which children with chronic abdominal pain are low suspicion for significant endoscopic findings. Clin Pediatr. 2019;58:79‐87. 10.1177/0009922818806317 30306797

