# Supplementary material for: Endoscopy and noninvasive tests in pediatric disorders of gut–brain interaction: A multicenter retrospective study of the Italian Society of Pediatric Gastroenterology, Hepatology, and Nutrition
Source: J Pediatr Gastroenterol Nutr. 2025 Jul 21;81(4):1089–99. doi: 10.1002/jpn3.70167 (PMC12484704; doi:10.1002/jpn3.70167)
Supplement: Supplementary file 1 — Supplementary fig. 1 Study design and population. [file JPN3-81-1089-s001.docx]

**Supplementary fig. 1 –** *Study design and population*

**
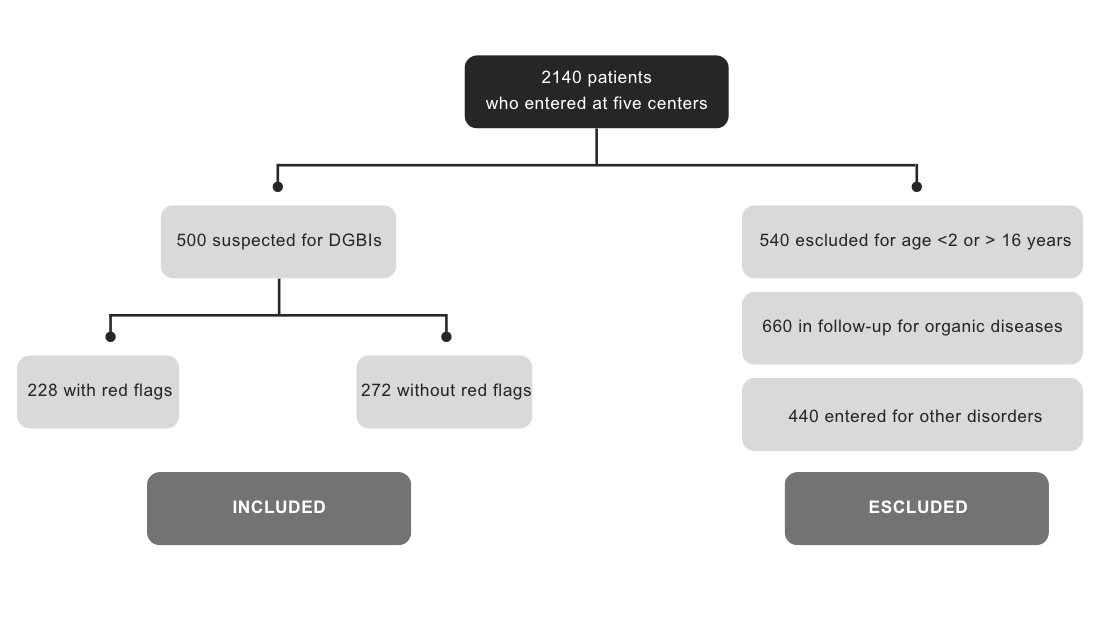
**

***Legend****: DGBIs = Disorders of Gut-Brain Interaction*
