# Supplementary material for: Endoscopy and noninvasive tests in pediatric disorders of gut–brain interaction: A multicenter retrospective study of the Italian Society of Pediatric Gastroenterology, Hepatology, and Nutrition
Source: J Pediatr Gastroenterol Nutr. 2025 Jul 21;81(4):1089–99. doi: 10.1002/jpn3.70167 (PMC12484704; doi:10.1002/jpn3.70167)
Supplement: Supplementary file 2 — Supplementary fig. 2 Univariate and multivariate analysis of the examined variables for predicting the presence of an organic disorder. [file JPN3-81-1089-s003.docx]

**Supplementary fig. 2** *– Univariate and multivariate analysis of the examined variables for predicting the presence of an organic disorder.*


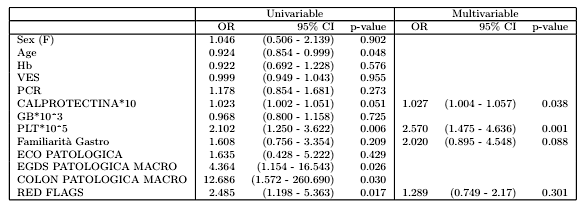


*Sex*

*Age*

*Hemoglobin (Hb)*

*Erythrocyte Sedimentation Rate (ERS) C-Reactive Protein (CRP)*

*Fecal Calprotectin (FC)*

*White Blood Cells (WBC)*

*Platelets (PLTs)*

*Family history positive for GI disease*

*Abnormal ultrasound*

*Abnormal EGD*

*Abnormal Ileo-colonscopy*

*Presence of red flags*
