# Supplementary material for: Endoscopy and noninvasive tests in pediatric disorders of gut–brain interaction: A multicenter retrospective study of the Italian Society of Pediatric Gastroenterology, Hepatology, and Nutrition
Source: J Pediatr Gastroenterol Nutr. 2025 Jul 21;81(4):1089–99. doi: 10.1002/jpn3.70167 (PMC12484704; doi:10.1002/jpn3.70167)
Supplement: Supplementary file 3 — Supplementary fig. 3 Comparison between groups with and without red flags for each laboratory test. [file JPN3-81-1089-s002.docx]

**Supplementary fig. 3 –** *Comparison between groups with and without red flags for each laboratory test*


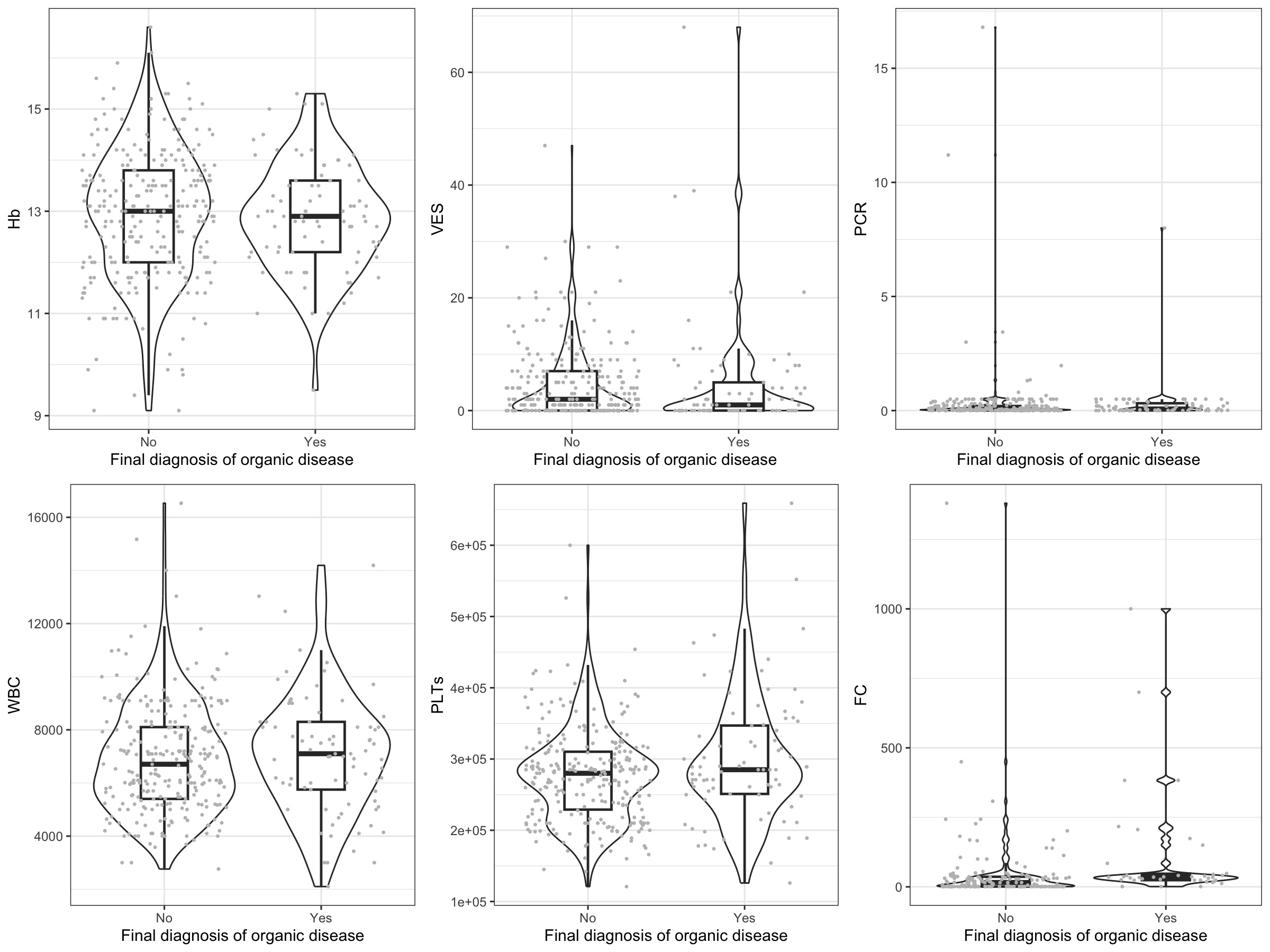


CRP

Hb

ESR

PLTs

FC

WBC

***Legend****: CRP = C-Reactive Protein, ESR = Eritrocite Sedimentation Rate, FC = Fecal Calprotectin, Hb = Hemoglobin, PLTs = Platelets, WBC = White Blood Cells.*
